# Evaluation of the diagnostic validity of the HIV VISITECT CD4 point-of-care rapid diagnostic test in Uganda

**DOI:** 10.1186/s12879-026-14021-y

**Published:** 2026-07-24

**Authors:** Oliver Nakamaanya, Bernard Bagaya, Willy Ssengooba, Erick Thokerunga, Henry Zamarano

**Affiliations:** 1 Department of Immunology and Molecular Biology, Makerere University, Kampala, Uganda; 2 Department of Medical Microbiology, Makerere University, Kampala, Uganda; 3 Makerere University Biomedical Research Center (MAKBRC), Kampala, Uganda; 4 Infectious Diseases Institute (IDI), Kampala, Uganda; 5 Uganda Protestant Medical Bureau, Kampala, Uganda

**Keywords:** CD4, VISITECT, PIMA, HIV, Advanced HIV disease, Point-of-care testing, Uganda, Diagnostic accuracy

## Abstract

**Background:**

HIV remains highly prevalent in Uganda, where CD4 T-cell counts are central to identifying and managing Advanced HIV Disease (AHD). Instrument-based point-of-care (POC) CD4 platforms face cost and operational constraints in resource-limited settings. The VISITECT CD4 lateral flow immunoassay (LFA) offers a non-instrument-based alternative, but its diagnostic validity against the Pima Analyzer had not been evaluated in Uganda.

**Objectives:**

To compare diagnostic validity of the VISITECT CD4 LFA against the Pima Analyzer at the 200 cells/µL AHD threshold and explore factors influencing performance.

**Methods:**

A comparative cross-sectional study was conducted between June and August 2023 at three Kampala health centres. Venous blood from 351 HIV-positive adults was tested simultaneously on both platforms. Sensitivity, specificity, PPV, NPV, and likelihood ratios were calculated with 95% CIs using the Pima Analyzer—a point-of-care, not gold-standard, platform—as reference; estimates reflect relative, not absolute, performance.

**Results:**

Most participants were female (68.11%), median age 31 years; mean CD4 count was 482 cells/µL (± 283.9) and AHD prevalence 22.79%. VISITECT CD4 LFA showed sensitivity of 86.25% (95% CI: 76.73–92.93), specificity of 90.41% (95% CI: 86.26–93.64), PPV of 72.63% (95% CI: 64.57–79.44), NPV of 95.70% (95% CI: 92.78–97.48), and inter-rater agreement κ = 0.957. No assessed factor significantly modified performance.

**Conclusions:**

The VISITECT CD4 LFA performed reliably as an AHD screening tool relative to the Pima Analyzer in resource-limited settings, particularly where instrument-based platforms are unavailable. It should complement, not replace, confirmatory testing; improvements in sensitivity and PPV remain warranted.

## Introduction

Human Immunodeficiency Virus (HIV) remains a highly prevalent disease worldwide, with developing countries such as Uganda disproportionately affected [1]. The epidemiological burden of HIV in sub-Saharan Africa (SSA) remains unacceptably high. In 2021, an estimated 38.4 million (31.1–43.9 million) people worldwide were living with HIV, with SSA accounting for 67% of that burden. Furthermore, SSA was responsible for 670,000 of the 1.5 million new infections and 280,000 of the 650,000 AIDS-related deaths worldwide in that year [2]. The Uganda Population-based HIV Impact Assessment found an HIV prevalence of 5.8% among people aged 15 years and above, with the prevalence varying by age category, geographical region, and population risk group [3].

CD4 T-cell count measurement is widely recommended to support and monitor HIV disease progression [4]. CD4 cells serve as a key indicator of immune function in HIV infection and remain pivotal to global and national guidelines as part of the battery of baseline investigations performed before initiating antiretroviral therapy (ART) [5, 6]. Presently, HIV management begins with conducting baseline investigations including CD4 cell count and viral load, among others [7, 8]. Whereas the World Health Organization (WHO) recommends viral load for monitoring HIV treatment response, patients with Advanced HIV Disease (AHD) require CD4 cell count to identify those with counts below 200 cells/mm³ who require screening for opportunistic infections, including cryptococcosis and mycobacterial disease [9–11]. CD4 cell counts are additionally important for detecting immunological treatment failure in patients already established on ART [8]. The Ugandan HIV/AIDS treatment guidelines integrate CD4 testing into routine HIV care and monitoring [12].

In Uganda, laboratory measurement of CD4 T-cell counts relies on point-of-care (POC) or near-POC analyzers, principally the Abbott Pima Analyzer (Abbott, Chicago, IL, USA) and BD FACSPresto (BD Biosciences, San Jose, CA, USA) [13, 14]. The Pima Analyzer is more user-friendly and has a more readily available supply chain on the local market [15]. Furthermore, as CD4 cell counts fall below the clinical threshold of 350 cells/µL, the accuracy of the Pima Analyzer improves [16]. Although conventional flow cytometry represents the established laboratory gold standard for CD4 enumeration, it is not available at the level of the health system for which the VISITECT CD4 is intended to be deployed [16–18]. The Pima Analyzer has been extensively validated against FACSCalibur flow cytometry in Ugandan settings, demonstrating high correlation coefficients (*r* = 0.943–0.948) and mean biases of approximately − 32.5 to − 48 cells/µL, providing a clinically meaningful comparator for this context [19]. Despite their clinical utility, instrument-based POC platforms are constrained by high repair and operational costs, power requirements, quality control demands, and the need for trained personnel [16, 17], barriers that have hindered CD4 testing in resource-limited and rural settings [18], and that underscore the need for simpler, non-instrument-based alternatives consistent with Uganda’s decentralization agenda under Vision 2030 [17].

The Omega VISITECT CD4 Advanced Disease test (VISITECT CD4; Omega Diagnostics, Scotland, UK) is one such tool. It is a lateral flow immunoassay (LFA)-based CD4 test that provides a semi-quantitative estimation of CD4 cell count at a threshold of 200 cells/µL [11]. As a rapid diagnostic test, the VISITECT CD4 is simpler to perform, less laborious, and requires no instrumentation [11]. Published data indicate an overall specificity of 95.0% and sensitivity of 81.9% for the VISITECT CD4 LFA [11]. To date, no study conducted in Uganda has compared the diagnostic performance of the VISITECT CD4 LFA to instrument-based POC platforms such as the Pima Analyzer, and the factors influencing its performance in this context remain unexplored. This study therefore aimed to compare the diagnostic validity of the VISITECT CD4 LFA with the Pima Analyzer and to identify factors influencing its performance in the Ugandan setting.

## Materials and methods

### Study design

A laboratory-based comparative cross-sectional study was conducted at Kisenyi Health Centre IV, Kawaala Health Centre IV, and Kiswa Health Centre IV between 1st June 2023 and 30th August 2023, to evaluate the diagnostic validity of the VISITECT CD4 LFA against the Pima Analyzer as the reference standard. The Pima Analyzer, an instrument-based POC platform, was used as the comparator rather than conventional flow cytometry (the laboratory gold standard for CD4 enumeration), which was not available at the study sites. The manuscript was prepared in accordance with the Standards for Reporting of Diagnostic Accuracy Studies (STARD) 2015 guidelines.

#### Inclusion criteria

Adult (≥ 18 years) HIV-positive individuals whose samples were submitted for CD4 analysis for AHD screening, ART initiation, or re-engagement in care; samples with adequate volume to complete all study assays; samples processed within 24 h of collection.

#### Exclusion criteria

Samples exceeding 24 h from the time of collection; samples with insufficient volume to complete the required testing procedures.

Advanced HIV Disease was defined as a CD4 cell count below 200 cells/mm³, consistent with WHO and Ugandan national guidelines.

### Study participants and eligibility

All health workers involved in CD4 analysis at the selected study sites were also recruited to assess inter-rater agreement and explore factors related to test performance. A total of 351 consecutive eligible participants were enrolled over the study period.

### Sample size

The sample size was calculated using the formula for estimating sensitivity of a diagnostic test, based on an expected sensitivity of 81.9% as reported in the literature for VISITECT CD4 LFA [11], a precision of 5%, and a 95% confidence level. Using the formula n = Z²P(1-P)/d², where Z = 1.96, *P* = 0.819, and d = 0.05, the minimum required sample size was 229. Accounting for an estimated 10% non-response and sample rejection rate, the target was set at 252 participants. The final enrolled sample of 351 exceeded this target, improving the precision of estimates.

### Data collection and laboratory methods

Data were collected using two structured questionnaires developed by the principal investigator. The first, administered to health workers, captured professional qualifications, training history, and experience with CD4 testing platforms. The second, completed through patient record review and direct interview, captured participant sociodemographic characteristics (age, sex), clinical information (reason for CD4 request, ART status, presence of opportunistic infections), and behavioural variables (tobacco smoking, alcohol intake). Both instruments were piloted prior to the main study to assess clarity and completeness.

At least 2 mL of whole venous blood was collected into ethylenediaminetetraacetic acid (EDTA) vacutainer tubes from each participant. Samples were run simultaneously on both the VISITECT CD4 LFA and the Pima Analyzer according to each manufacturer’s standard operating procedures (SOPs) [20, 21].

For the VISITECT CD4 LFA, visual interpretation of each test result was performed independently by two blinded technicians or healthcare workers. Disagreements in result interpretation were resolved by involving the principal investigator. Inter-rater agreement between the two independent readers was assessed using Cohen’s kappa. For the Pima Analyzer, samples were processed according to the device manufacturer’s instructions [20, 21]. Results from all procedures were recorded and entered into a Microsoft Excel spreadsheet for subsequent analysis.

### Data analysis

Data were cleaned, coded, and imported into STATA version 17 (StataCorp, College Station, TX, USA) for analysis. Sociodemographic data were summarised as median with interquartile range (IQR) or mean with standard deviation (SD) for continuous variables, and as frequencies with percentages for categorical variables. CD4 count results from the Pima Analyzer were evaluated at the recommended AHD cut-off of 200 cells/µL against VISITECT CD4 LFA results.

The sensitivity, specificity, PPV, NPV, positive and negative likelihood ratios (LHR+, LHR−), and overall accuracy of the VISITECT CD4 LFA were calculated and expressed as percentages with corresponding 95% confidence intervals, using the Pima Analyzer as the reference standard.

Factors potentially influencing diagnostic agreement between the VISITECT CD4 LFA and the Pima Analyzer — including presence of opportunistic infections, tobacco smoking, and alcohol intake — were assessed using Pearson’s chi-square test. For cells in which the expected frequency was below 5, Fisher’s exact test was applied. A p-value of less than 0.05 was considered statistically significant.

There were no missing values for the primary outcome variables (VISITECT CD4 LFA and Pima Analyzer results) or the sociodemographic and clinical variables reported in this analysis; all 351 participants had complete data for the variables assessed. Multivariable regression modelling was not undertaken, as the assessment of factors influencing diagnostic agreement was an exploratory secondary objective and several subgroups (e.g., specific opportunistic infections) contained too few events to support stable multivariable estimation; unadjusted bivariate comparisons using chi-square and Fisher’s exact tests were therefore considered the more appropriate and transparent approach for this exploratory analysis.

Inter-rater agreement between the two independent VISITECT result readers was assessed using the Cohen’s kappa statistic (κ), calculated as:

$$\kappa {\rm{ }} = {\rm{ }}\left( {Po{\rm{ }} - {\rm{ }}Pe} \right){\rm{ }}/{\rm{ }}\left( {1{\rm{ }} - {\rm{ }}Pe} \right)$$

where Po is the observed proportional agreement and Pe is the hypothetical probability of chance agreement. Agreement was interpreted as: poor (κ < 0.20), fair (0.21–0.40), moderate (0.41–0.60), substantial (0.61–0.80), near-perfect (0.81–0.99), or perfect (1.00).

### Ethics

This research was conducted in accordance with the Declaration of Helsinki and approved by the Makerere University School of Biomedical Sciences Research Ethics Committee (Approval number: SBS-2023-441). All participants provided informed consent prior to their involvement in the study.

## Results

### Participant flow

Of 380 participants assessed for eligibility during the study period, 351 met the inclusion criteria and were enrolled. Twenty-nine were excluded: 17 had samples with insufficient volume and 12 had samples exceeding the 24-hour processing window. All 351 enrolled participants had valid results on both the Pima Analyzer and the VISITECT CD4 LFA and were included in the final analysis.

### Sociodemographic and clinical characteristics

Of the 351 participants, the majority were female (239/351, 68.11%) and 112 (32.00%) were male. The median age was 31 years (IQR 25–38). The mean CD4 count by Pima Analyzer was 482 cells/µL (SD ± 283.9). The prevalence of AHD, defined as a CD4 count of ≤ 200 cells/µL by Pima Analyzer, was 22.79% (80/351) (Table 1).

**Table 1:** Demographic and CD4 count characteristics of study participants, stratified by AHD status on Pima Analyzer

Characteristic	Overall (*n* = 351)	PIMA ≤ 200 cells/µL (*n* = 80)	PIMA > 200 cells/µL (*n* = 271)
Female, n (%)	239 (68.11%)	47 (19.75%)	192 (80.33%)
Male, n (%)	112 (32.00%)	33 (29.46%)	79 (70.54%)
Age, years, median (IQR)	31 (25–38)	31 (27–38)	31 (24–38)
Mean CD4 count, Pima cells/µL (SD)	482 (± 283.9)	107 (± 63.1)	531 (± 248)

### Diagnostic performance of VISITECT CD4 LFA

Of 351 participants, 95 were classified as AHD-positive (CD4 ≤ 200 cells/µL) by the VISITECT CD4 LFA and 256 as AHD-negative. Using the Pima Analyzer as the reference standard, there were 69 true positives, 245 true negatives, 11 false negatives, and 26 false positives. The cross-tabulation and derived diagnostic performance characteristics are presented in Table 2.

At the recommended AHD threshold of ≤ 200 CD4 cells/µL, the VISITECT CD4 LFA demonstrated a sensitivity of 86.25% (95% CI: 76.73–92.93) and a specificity of 90.41% (95% CI: 86.26–93.64). The PPV was 72.63% (95% CI: 64.57–79.44) and the NPV was 95.70% (95% CI: 92.78–97.48). The positive likelihood ratio was 8.99 (95% CI: 6.17–13.09) and the negative likelihood ratio was 0.15 (95% CI: 0.09–0.26). Overall accuracy was 89.46% (95% CI: 85.76–92.47).

**Table 2:** VISITECT CD4 LFA performance characteristics at the ≤ 200 cells/µL AHD threshold using the Pima Analyzer as reference standard

		Pima ≤ 200 cells/µL (AHD+)	Pima > 200 cells/µL (AHD−)	Total
VISITECT	**≤ 200 cells/µL (AHD+)**	69	26	95
	**> 200 cells/µL (AHD−)**	11	245	256
Total		**80**	**271**	**351**
Sensitivity %, (95% CI)				86.25 (76.73–92.93)
Specificity %, (95% CI)				90.41 (86.26–93.64)
PPV %, (95% CI)				72.63 (64.57–79.44)
NPV %, (95% CI)				95.70 (92.78–97.48)
LHR+ (95% CI)				8.99 (6.17–13.09)
LHR− (95% CI)				0.15 (0.09–0.26)
Accuracy %, (95% CI)				89.46 (85.76–92.47)
Prevalence				22.79%

### Inter-rater agreement

The overall inter-rater agreement between the two independent readers for VISITECT CD4 LFA result interpretation was 95.7%, corresponding to a Cohen’s κ of 0.957, indicating near-perfect agreement.

### Reasons for CD4 count request

**Fig. 1:**
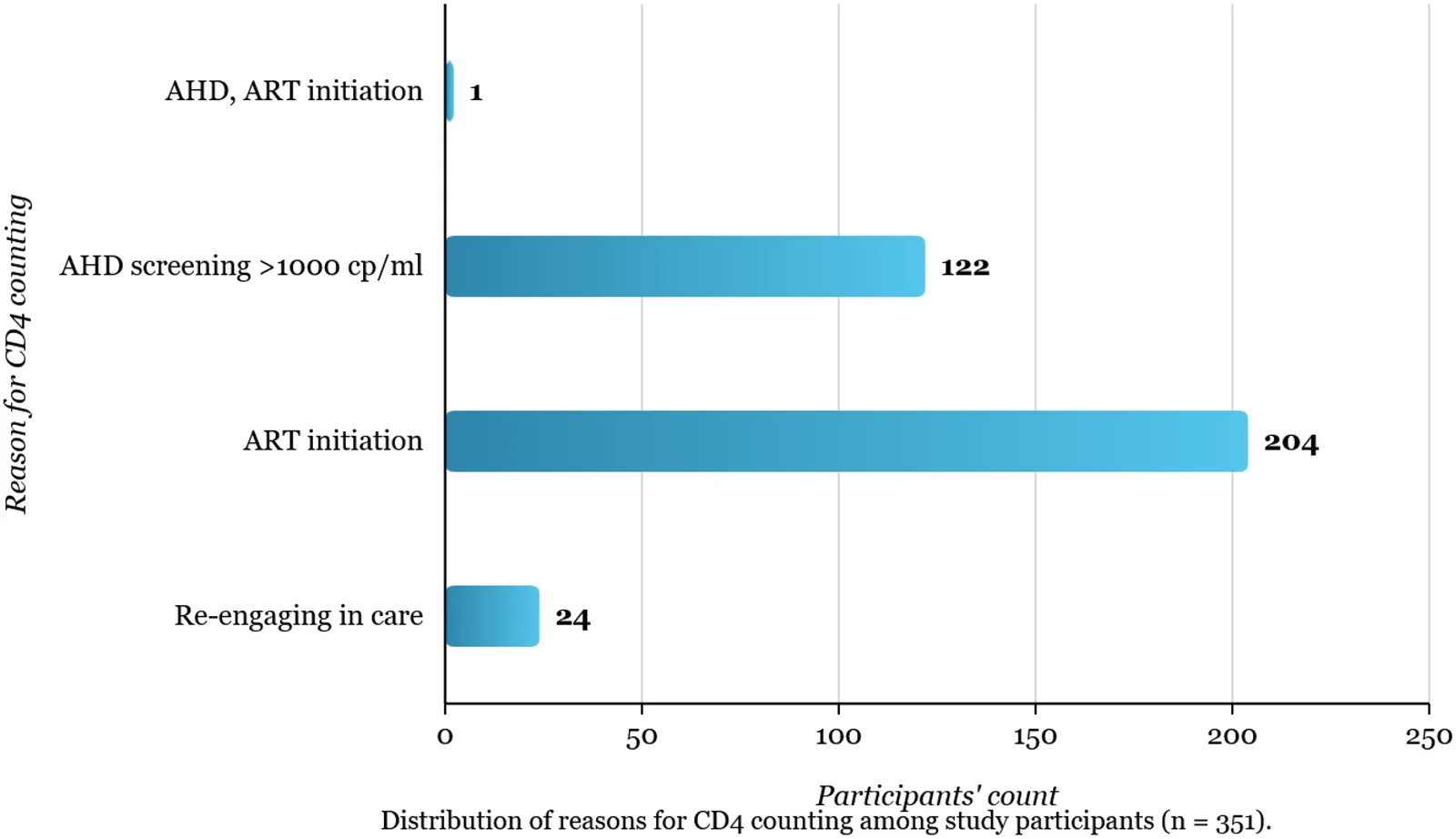
Number of participants based on reason for CD4 + cell counting

**Fig. 2:**
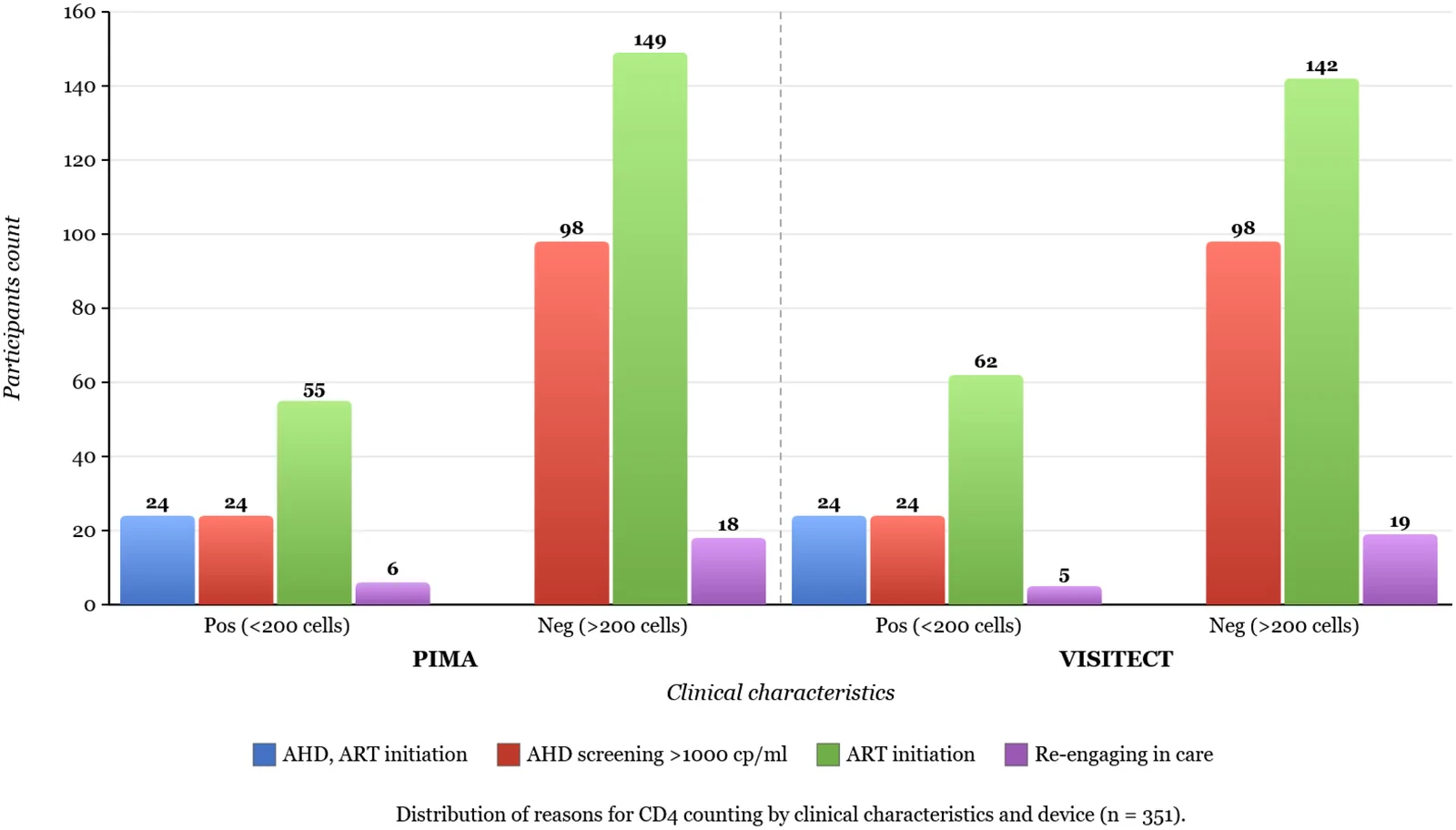
Number of participants based on CD4 cell counts and reasons for testing

Overall, the majority of participants (204/351, 58.12%) were tested as a requirement for ART initiation, followed by clinical suspicion of AHD (122/351, 34.76%) (Fig. 1). Among those being tested to initiate ART, the VISITECT CD4 LFA detected AHD in 62/204 (30.4%) participants compared with 55/204 (27.0%) by Pima Analyzer, representing a 3.4%-point overestimation of AHD by VISITECT. Among participants tested due to clinical suspicion of AHD or for re-engagement in care, the VISITECT CD4 LFA and Pima Analyzer produced comparable results (Fig. 2).

### Factors influencing diagnostic validity

All three study laboratories had environmental monitoring systems in place (temperature and humidity), with conditions remaining within acceptable ranges throughout the study period. All laboratories were participating in external quality assurance (EQA) programmes, with pass rates of 8–9 out of 10. All participating health workers held requisite professional qualifications and had received in-house training on the VISITECT CD4 LFA from the principal investigator. All samples were venous blood collected in EDTA tubes.

These analyses were exploratory and unadjusted, intended to generate hypotheses rather than to provide definitive effect estimates, and should be interpreted with caution given the small numbers of participants in several subgroups. The results of the factor analysis are presented in Table 3. The presence of opportunistic infections, tobacco smoking, and alcohol intake were each assessed for their effect on agreement between VISITECT CD4 LFA and Pima Analyzer results. None of the factors examined was a statistically significant modifier of diagnostic agreement (all *p* > 0.05). Note that for sub-groups with expected cell frequencies below 5 (Candidiasis, Cryptococcal infection, Pneumonia), Fisher’s exact test was used in place of chi-square.

**Table 3:** Analysis of factors affecting diagnostic agreement between VISITECT CD4 LFA and Pima Analyzer results

Factor	Agreement: Yes, *n* (%)	Agreement: No, *n* (%)	Test statistic	*P* value
**Opportunistic infection**			χ² = 10.128*	0.072
Candidiasis	6 (100.0)	0 (0.0)		
Cryptococcal infection	5 (100.0)	0 (0.0)		
Hypertension	0 (0.0)	1 (100.0)		
Tuberculosis	18 (90.0)	2 (10.0)		
Pneumonia	1 (100.0)	0 (0.0)		
None	285 (89.62)	33 (10.38)		
**Smoking**			χ² = 0.936	0.333
Yes	8 (100.0)	0 (0.0)		
No	307 (89.5)	36 (10.5)		
**Alcohol intake**			χ² = 2.008	0.156
Yes	44 (95.65)	2 (4.35)		
No	271 (88.85)	34 (11.15)		

*Fisher’s exact test applied for sub-groups with expected cell frequency < 5 (Candidiasis, Cryptococcal infection, Pneumonia). Agreement is defined as concordance between VISITECT CD4 LFA and Pima Analyzer classification at the ≤ 200 cells/µL threshold

## Discussion

This study assessed the diagnostic validity of the VISITECT CD4 LFA against the Pima Analyzer for AHD screening at the 200 cells/µL threshold among HIV-positive adults attending public health facilities in Kampala, Uganda.

These findings contrast with those of Ndlovu et al. [11], who reported a sensitivity of 98.3% and a specificity of 77.2% for the VISITECT CD4 LFA compared with the Pima Analyzer using finger-prick blood. The sensitivity and specificity in the present study are thus inverted relative to those of Ndlovu et al., with lower sensitivity but substantially higher specificity. This pattern is broadly consistent with the wider literature on VISITECT CD4 LFA performance, which demonstrates higher specificity but lower sensitivity when venous rather than finger-prick blood is used [12, 13]. These differences may reflect the volume and cellular composition of the sample delivered to the LFA membrane, differences in AHD prevalence between study populations, and variation in VISITECT CD4 LFA performance under different environmental and operational conditions. Low disease prevalence is well established as a determinant of PPV and NPV, and at the prevalence observed in this study, a moderate PPV is consistent with expected test performance even for assays with high intrinsic specificity [22].

Regarding the choice of reference standard, conventional flow cytometry represents the established gold standard for CD4 enumeration. The Pima Analyzer was used as the reference rather than flow cytometry, reflecting both logistical constraints at the study sites and the operational context in which the VISITECT CD4 LFA is intended to be deployed. This is an acknowledged limitation, as the performance characteristics reported here represent validity relative to a POC reference rather than an absolute laboratory standard. Importantly, the Pima Analyzer has itself been extensively validated against FACSCalibur flow cytometry in Ugandan settings, demonstrating high correlation and mean biases within a clinically acceptable range [23, 24], which supports its use as a meaningful comparator for the health system context in which this test would be operationalized. Because any misclassification by the Pima Analyzer relative to flow cytometry is carried directly into the calculation of VISITECT CD4 LFA performance, the sensitivity and specificity estimates reported here should be interpreted as measures of relative, rather than absolute, diagnostic validity, and may differ from estimates that would be obtained against a flow cytometry reference.

The false-negative cases identified in this study carry direct clinical consequences. Individuals with CD4 counts below 200 cells/mm³ who are misclassified as non-AHD will not receive the AHD care package, including cryptococcal antigen screening, tuberculosis prophylaxis, and cotrimoxazole preventive therapy, all of which are associated with reduced morbidity and mortality [25–27]. This is not a trivial concern in a setting where AHD-related complications remain a leading cause of HIV-associated death. While the sensitivity observed may be acceptable for a screening tool in the context of expanded access at peripheral facilities, it falls short of what would be required for a standalone gating test, and efforts to improve sensitivity through sample type optimisation or threshold recalibration remain warranted.

The false-positive results present a different but equally important operational challenge. In a resource-constrained health system, unnecessary initiation of the AHD care package carries real costs, both financial and in terms of patient anxiety. This reinforces the case for confirmatory testing with an instrument-based platform where feasible, particularly before commencing intensive opportunistic infection prophylaxis or treatment.

The high NPV is the most operationally reassuring finding of this study. In a screening context, a negative VISITECT CD4 LFA result carries a high probability of truly ruling out AHD, a property of particular value in peripheral facilities where the consequences of over-referral are also significant. The positive likelihood ratio indicates that a positive VISITECT result substantially increases the post-test probability of AHD, while the negative likelihood ratio confirms meaningful reduction in post-test probability when the result is negative, together supporting its use as a triage instrument.

In exploratory, unadjusted analyses, none of the factors examined — opportunistic infections, smoking, and alcohol intake — significantly modified the agreement between VISITECT CD4 LFA and Pima Analyzer. Given the small subgroup sizes for several categories and the absence of adjustment for confounding, these findings are best regarded as hypothesis-generating rather than conclusive, and do not rule out clinically meaningful effects that a larger, adequately powered study might detect. The near-perfect inter-rater agreement confirms that the VISITECT CD4 LFA can be reliably interpreted by trained health workers, which is a practical prerequisite for decentralized deployment. The consistent EQA performance and appropriate environmental conditions across all three study sites further strengthen confidence in the internal validity of these results.

Study Limitations: The primary limitation of this study is the use of the Pima Analyzer rather than conventional flow cytometry as the reference standard, which may result in some degree of reference standard misclassification. The study was conducted in three urban health centres in Kampala and the findings may not be directly generalizable to rural or peri-urban settings with different patient profiles, ambient conditions, or supply chain characteristics. The predominance of female participants (68.1%) in this sample is broadly consistent with patterns of higher health-facility attendance among women within Uganda’s HIV programme, but the study population was drawn exclusively from urban public health centres and may under-represent men, adolescents, and individuals in rural or peri-urban areas, who together make up a substantial share of people living with HIV nationally; caution is therefore warranted in extrapolating these findings beyond similar urban clinical settings. Sub-group analysis of the effect of opportunistic infections was limited by small cell sizes for several infection categories, which restricted statistical power despite the use of Fisher’s exact test. The cross-sectional design precludes longitudinal assessment of test performance across a range of clinical trajectories.

## Conclusion

The VISITECT CD4 LFA demonstrated reliable diagnostic performance for AHD screening at the 200 cells/µL threshold relative to the Pima Analyzer, an instrument-based point-of-care comparator rather than the laboratory gold standard; in exploratory, unadjusted analyses, its performance was not significantly modified by opportunistic infections, tobacco smoking, or alcohol intake. The near-perfect inter-rater agreement confirms that trained health workers can reliably interpret results, a prerequisite for decentralized deployment, and its high NPV is particularly valuable in peripheral settings, where confidently ruling out AHD reduces unnecessary referral and supports rational use of limited resources.

These findings support the VISITECT CD4 LFA as a screening tool for AHD in resource-limited settings, particularly where instrument-based platforms are unavailable, rather than as a replacement for confirmatory testing. Given the false-negative rate observed, confirmatory testing with an instrument-based platform remains advisable before committing a patient to the full AHD care package. Further work to optimize its performance with venous blood and to reduce false positives would strengthen its role within routine AHD screening algorithms at lower-level health facilities.

## Data Availability

The dataset supporting the conclusions of this article is available from the corresponding author on reasonable request in accordance with Makerere University data sharing policies and applicable ethics committee approvals.
